# Long noncoding RNA AFAP1-AS1 predicts a poor prognosis and regulates non–small cell lung cancer cell proliferation by epigenetically repressing p21 expression

**DOI:** 10.1186/s12943-018-0836-7

**Published:** 2018-05-24

**Authors:** Dandan Yin, Xiyi Lu, Jun Su, Xuezhi He, Wei De, Jinsong Yang, Wei Li, Liang Han, Erbao Zhang

**Affiliations:** 1 Cancer Research and Biotherapy Center, the Second Affiliated Hospital of Southeast University, Nanjing, Jiangsu People’s Republic of China; 2 Department of Oncology, First Affiliated Hospital of Nanjing Medical University, Nanjing, Jiangsu People’s Republic of China; 3 Department of Oncology, the Affiliated Jiangyin Hospital of Southeast University Medical College, Jiangyin, Jiangsu China; 4 Department of Biochemistry and Molecular Biology, Nanjing Medical University, Nanjing, Jiangsu People’s Republic of China; 5 Department of Oncology, Nanjing First Hospital, Nanjing Medical University, Nanjing, Jiangsu People’s Republic of China; 6 Department of Oncology, Xuzhou Central Hospital, Affiliated Xuzhou Hospital, College of Medicine, Southeast University, Xuzhou, Jiangsu People’s Republic of China; 7 Department of Epidemiology and Biostatistics, Jiangsu Key Lab of Cancer Biomarkers, Prevention and Treatment, Collaborative Innovation Center for Cancer Personalized Medicine, School of Public Health, Nanjing Medical University, Nanjing, People’s Republic of China

**Keywords:** AFAP1-AS1, Cell proliferation, EZH2, p21, NSCLC

## Abstract

**Background:**

Mounting evidence indicates that long noncoding RNAs (lncRNAs) could play a pivotal role in cancer biology. However, the role and molecular mechanism and global genes that were mediated by lncRNA AFAP1-AS1 in non–small cell lung cancer (NSCLC) remain largely unknown.

**Methods:**

Expression of AFAP1-AS1 was analyzed in 92 NSCLC tissues and cell lines by Quantitative real time polymerase chain reaction (qRT-PCR). The effect of AFAP1-AS1 on proliferation was evaluated by function assays both in in vitro and in vivo. RNA-seq assays were performed after knockdown AFAP1-AS1. RNA immunoprecipitation (RIP) was performed to confirm the interaction between AFAP1-AS1 and EZH2. Chromatin immunoprecipitation (ChIP) was used to study the promoter region of p21.

**Results:**

AFAP1-AS1 expression was increased in NSCLC tissues and was correlated with clinical outcomes of NSCLC. Further experiments revealed that inhibition of its expression in NSCLC cells resulted in diminished cell growth in vitro and in vivo. RNA-seq revealed that knockdown of AFAP1-AS1 could induce the expression of p21. Mechanistic investigations found that AFAP1-AS1 could interact with EZH2 and recruit EZH2 to the promoter regions of p21, thus epigenetically repressing p21 expression.

**Conclusions:**

Together, these results suggest that lncRNA AFAP1-AS1 may serve as a candidate prognostic biomarker and target for new therapies in human NSCLC.

**Electronic supplementary material:**

The online version of this article (10.1186/s12943-018-0836-7) contains supplementary material, which is available to authorized users.

## Background

Lung cancer is the most frequent cause of cancer-related death in worldwide [1, 2]. Non-small cell lung cancer (NSCLC) accounts for approximately 85% of all lung cancer newly cases and can be categorized into two common subtypes, squamous cell carcinoma, adenocarcinoma and large cell carcinoma [3]. Even though current advances in the chemotherapy and molecular targeting therapy for NSCLC, the overall 5-year survival rate for the disease is less than 15% due to the limited therapeutic options, tumor metastasis, and recurrence [4]. Undoubtedly, better understanding of the carcinogenesis is critical for the advance of diagnostic markers and aid novel effective therapies for NSCLC patients.

In recent years, it has become increasingly apparent that the noncoding portion of the genome is of crucial functional importance in both normal physiology and diseases [5]. Long non-coding RNAs (lncRNAs) are classified as a new kind of non-coding RNA (ncRNA) that is longer than 200 nucleotides in length with no or limited protein-coding potential [6, 7]. Increasing studies have shown that lncRNAs are pervasively involved in many biological processes, including cellular development, differentiation, apoptosis, inflammation, autophagy, and cancer [8–12]. In addition, an emerging paradigm of the aberrant lncRNAs has been found to participate in NSCLC development and progression. For example, the lncRNA MALAT1 is a highly conserved nuclear ncRNA and a predictive marker for metastasis development in lung cancer [13]. Elevated LINC00473 expression correlates with poor prognosis, and elevated LINC00473 serve as a robust biomarker for LKB1-inactivated NSCLC [14]. In this regard, identifying the associated molecular mechanisms of cancer-associated lncRNAs is necessary for understanding progression and establishing better treatment of NSCLC.

LncRNA actin filament-associated protein 1 antisense RNA 1 (AFAP1-AS1), a 6.8-kb lncRNA that is located in the chromosome 4p16.1, was initially identified by Wu et al. and was extremely hypomethylated and overexpressed in Barrett esophagus (BE) and esophageal adenocarcinoma (EAC) tissues and cells [15]. In addition, recently studies showed that increased expression of AFAP1-AS1 could participate in the progression of a variety of tumors, including nasopharyngeal carcinoma, pancreatic ductal adenocarcinoma, hepatocellular carcinoma, colorectal cancer and gallbladder cancer [16–20]. These results indicate that AFAP1-AS1 may be necessary for development, and that its dysregulation may participate in human cancer progression. Although, Zhang et al. found that knockdown of AFAP1-AS1 inhibits tumor cell growth and invasion in lung cancer [21]. However, the biological functions of AFAP1-AS1 in the control of NSCLC tumorigenesis have not been well characterized. Especially, the molecular mechanism and global genes that were mediated by AFAP1-AS1 had not yet been established. These prompted us to explore the role of AFAP1-AS1 in human NSCLC.

In this study, we investigated the potential molecular mechanisms of AFAP1-AS1 on NSCLC progression. We found that AFAP1-AS1 was significantly upregulated in NSCLC tissues compared with normal lung tissues, and may serve as an independent predictor for the overall survival in NSCLC. In addition, AFAP1-AS1 could regulate cell proliferation both in vitro and in vivo. Further experiments demonstrated that AFAP1-AS1 was associated with EZH2 and that this association was required for the epigenetic repression of p21, which encodes a potent cyclin-dependent protein kinase inhibitor (CKI), thus contributing to the regulation of both the NSCLC cell cycle and proliferation. Taken together, the findings may provide new insights into the critical role of the lncRNA AFAP1-AS1 in human NSCLC tumorigenesis.

## Methods

### Tissue collection and ethics statement

We obtained paired NSCLC and adjacent normal lung tissues from 92 patients who underwent primary surgical resection in the Department of Thoracic Surgery, zhongda Hospital, Southeast University School of Medicine, China. No patient had received local or systemic treatment before any operation. All collected tissue samples were immersed in RNA Later stabilization solution (Qiagen) and were immediately frozen in liquid nitrogen and stored at − 80 °C until RNA isolation. The clinicopathologic characteristics of the patients with NSCLC were summarized in Table 1. Our study protocol was approved by the Institutional Review Board of Southeast University, and all of the participants signed an informed consent form.

**Table 1:** The clinic-pathological factors of 92 NSCLC patients

Characteristics	Numbers	Expression of AFAP1-AS1		*p* value*
		low(N = 46)	high(N = 46)	
Sex				0.402
male	42	23	19	
female	50	23	27	
Age				0.835
≤60	45	22	23	
> 60	47	24	23	
Histological grade				0.179
Middle or low	75	35	40	
high	17	11	6	
Histological classification				0.532
SCC (Squamous cell carcinoma)	47	25	22	
AD (adenocarcinoma or other)	45	21	24	
TNM stage				0.182
Iand II	62	34	28	
III and IV	30	12	18	
Lymph node metastasis				0.529
negative	41	22	19	
positive	51	24	27	
Tumor size				< 0.001**
≤3 cm	36	28	8	
> 3 cm	56	18	38	
History of smoking				0.144
Ever	49	28	21	
Never	43	18	25	

Chi-square test

**p < 0.001

### Cell culture

Cell lines (16HBE, A549, SPC-A1 and H1299) were purchased from the Institute of Biochemistry and Cell Biology of the Chinese Academy of Sciences (Shanghai, China). Cells were maintained in RPMI Medium 1640 basic media (GIBCOBRL, Invitrogen, Carlsbad, CA), and (DMEM; GIBCO-BRL, Invitrogen) in a humidified incubator at 37 °C with 5% CO2. All of the media used was supplemented with heat-inactivated 10% fetal bovine serum (FBS) and antibiotics (100 U/ml penicillin and 100 mg/ml streptomycin) (Invitrogen).

### RNA extraction and qRT-PCR analyses

The total RNA was extracted from tissues or cultured cells with TRIzol reagent (Invitrogen), according to the manufacturer’s protocol. One microgram total RNA was reverse transcribed using PrimeScript RT Reagent Kit with gDNA Eraser (Takara). cDNA was used for subsequent qRT-PCR reactions (SYBR, TaKaRa) according to the manufacturer’s instructions. The results were normalized to the expression of GAPDH. The qRT-PCR and data collection were carried out on ABI 7500 real-time PCR system (Applied Biosystems, Foster City, CA, USA). The primer sequences are summarized in Additional file 1: Table S1.

### Subcellular fractionation location

The separation of the nuclear and cytosolic fractions was performed using the PARIS Kit (Life Technologies, Carlsbad, CA, USA) according to the manufacturer’s instructions.

### Transfection of cell lines

NSCLC cell lines were transfected with specific siRNA oligonucleotides. Typically, cells were seeded at six-well plates and then transfected the next day with specific siRNA (100 nM) and control siRNA (100 nM) by using Lipofectamine 2000, according to the manufacturer’s protocol (Invitrogen). The sequences for siRNAs were listed in Additional file 1: Table S1.

### Cell proliferation assays

Cell proliferation was monitored using Cell Proliferation Reagent Kit I (MTT) (3-(4,5-dimethyl-2-thiazolyl)-2,5-diphenyl-2-H-tetrazolium bromide) (Roche, Basel, Switzerland). The transfected cells were plated in 96-well plates (3000 cells/well). Cell proliferation was determined every 24 h following the manufacturer’s protocol. For the colony-formation assay, a certain number of transfected cells were placed into each well of a six-well plate and maintained in media containing 10% FBS for 2 weeks, replacing the medium every 4 days. Colonies were fixed with methanol and stained with 0.1% crystal violet (Sigma-Aldrich, St. Louis, MO, USA) in PBS for 15 min. The colony formation was determined by counting the number of stained colonies. Triplicate wells were measured in each treatment group. BrdU experiments were performed using a BrdU Cell Proliferation Assay Kit (Millipore, Cat.No.2750) according to the manufacturer’s instructions. The higher OD reading represents the higher BrdU concentration in the sample.

### Flow cytometric analysis

Cells for cell-cycle analysis were stained with propidium oxide by the CycleTEST PLUS DNA Reagent Kit (BD Biosciences) following the protocol and analyzed by FACScan. The percentage of the cells in G0–G1, S, and G2–M phase were counted and compared.

### Xenograft study

A549 cells were stably transfected with shRNA and empty vector and harvested from cell culture plates, then cells were xenografted into BALB/c male nude mice. The tumor volumes and weights were measured every three days in mice; the tumor volumes were measured as length×width^2^ × 0.5. Twenty-one days after injection, the mice were killed and tumor weights were measured and used for further analysis. Immunohistochemistry was performed as previously described [22]. Anti-Ki-67 was from Abcam.

### Whole transcriptome deep sequencing

Total RNA from the A549 cells with AFAP1-AS1 knockdown and control A549 cells were isolated and quantified. The complementary DNA (cDNA) libraries for single-end sequencing were prepared using Ion Total RNA-Seq Kit v2.0 (Life Technologies) according to the manufacturer’s instructions. The cDNA libraries were then processed for the Proton Sequencing process according to the commercially available protocols. Samples were diluted and mixed, the mixture was processed on a OneTouch 2 instrument (Life Technologies) and enriched on a OneTouch 2 ES station (Life Technologies) for preparing the template-positive Ion PI™ Ion Sphere™ Particles (Life Technologies) according to Ion PI™ Template OT2 200 Kit v2.0 (Life Technologies). After enrichment, the mixed template-positive Ion PI™ Ion Sphere™ Particles of samples was loaded on to 1 P1v2 Proton Chip (Life Technologies) and sequenced on Proton Sequencers according to Ion PI Sequencing 200 Kit v2.0 (Life Technologies) by NovelBio Corp. Laboratory, Shanghai. Data are available in Additional file 2: Table S2.

### Western blot assay

The cells were lysed using mammalian protein extraction reagent RIPA (Beyotime, Haimen, China) supplemented with protease inhibitors cocktail (Roche) and PMSF (Roche). Fifty micrograms of the protein extractions were separated by 10% SDS-PAGE transferred to 0.22 mm nitrocellulose (NC) membranes (Sigma-Aldrich) and incubated with specific antibodies.The autoradiograms were quantified by densitometry (Quantity One software; Anti-p21 was from Abcam. Results were normalized to the expression of GAPDH.

### RNA immunoprecipitation

RNA immunoprecipitation (RIP) experiments were performed using a Magna RIP RNA-Binding Protein Immunoprecipitation Kit (Millipore) according to the manufacturer’s instructions. Antibodies for RIP assays against EZH2 and SUZ12 were purchased from Abcam.

### ChIP assays

The ChIP assays were performed using the EZ-CHIP KIT according to the manufacturer’s instructions (Millipore, Billerica, MA, USA). EZH2 and SUZ12 antibodies were obtained from Abcam (Hercules, CA, USA). H3 trimethyl Lys 27 antibody was purchased from Millipore. Quantification of immunoprecipitated DNA was performed using qPCR with SYBR Green Mix (Takara). The ChIP data were calculated as a percentage relative to the input DNA using the eq. 2^[Input Ct- Target Ct]^ × 100 (%).

### Statistical analysis

Statistical analysis was performed using the SPSS software package (version 17.0, SPSS Inc., Armonk, NY, USA) and GraphPad Prism 5 (GraphPad Software, La Jolla, CA, USA). Statistical significance was tested by a Student’s t-test or a Chi-square test as appropriate. Survival analysis was performed using the Kaplan–Meier method, and the log-rank test was used to compare the differences between patient groups. Statistics with *P*-value < 0.05 were considered as statistically significant.

## Results

### AFAP1-AS1 is up-regulated in NSCLC tumor tissues and associated with TNM stage and tumor size

**Fig. 1:**
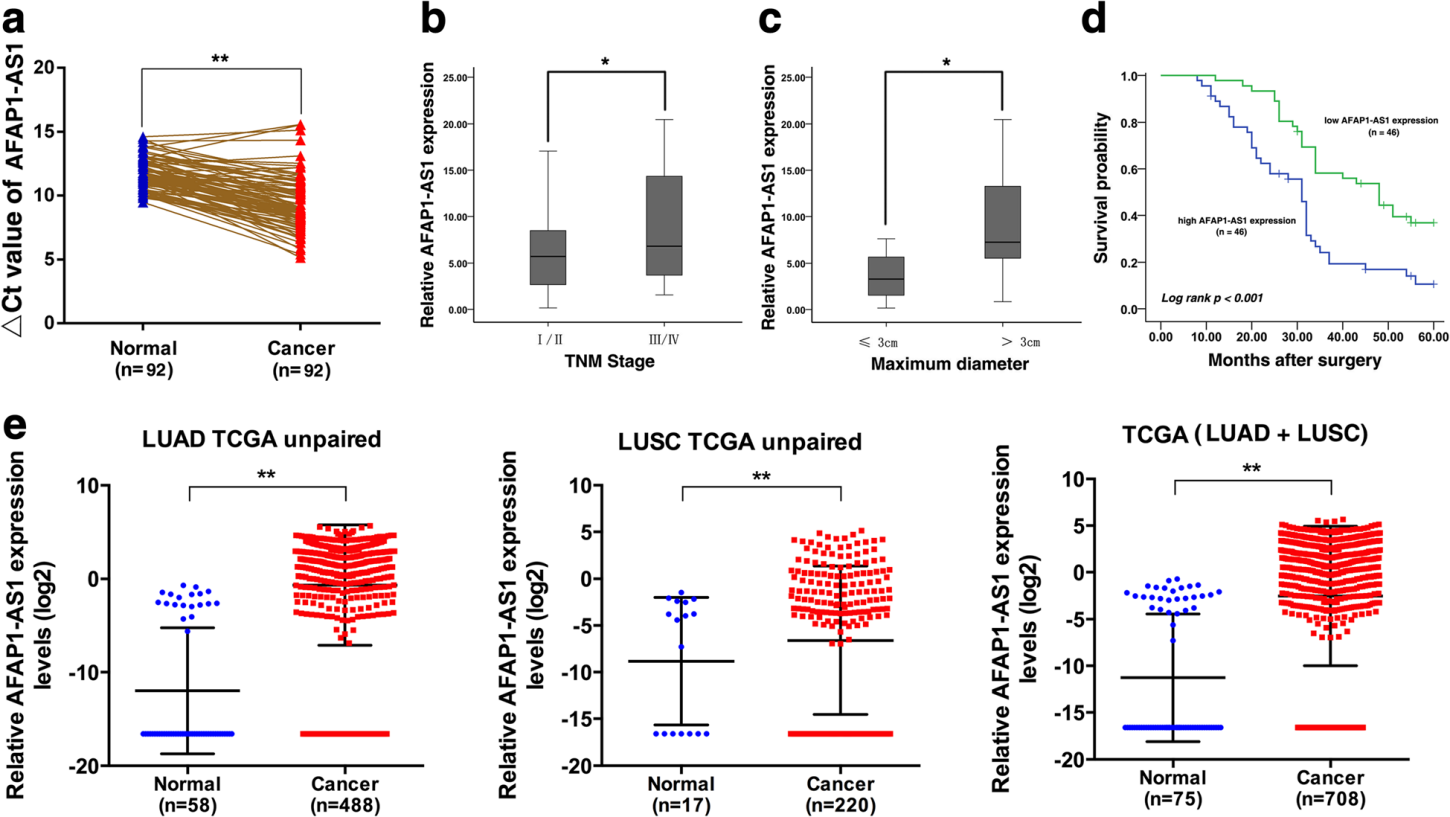
Relative AFAP1-AS1 expression in NSCLC tissues and its clinical significance. **a** Relative expression levels of AFAP1-AS1 in NSCLC tissues (*n* = 92) compared with corresponding non-tumor tissues (n = 92). AFAP1-AS1 expression was examined using qPCR and was normalized to GAPDH expression. The △Ct value was determined by subtracting the GAPDH Ct value from the AFAP1-AS1 Ct value Smaller △Ct values indicate higher expression. **b** AFAP1-AS1 expression was significantly higher in patients with higher pathological stages (III/IV) than in those with lower pathological stages (I/II). **c** AFAP1-AS1 expression was significantly higher in patients with larger tumor sizes (> 3 cm) than in those with smaller tumor sizes (≤3 cm). **d** Kaplan-Meier overall survival (OS) curves according to AFAP1-AS1 expression levels. **e** AFAP1-AS1 expression in NSCLC tissues in TCGA database. *, *P* < 0.05, and **, *P* < 0.01

First, Q-PCR analysis was perfomed to investigate AFAP1-AS1 expression in 92 pairs of NSCLC tumor tissues compared with adjacent normal tissues. As shown in Fig. 1a, AFAP1-AS1 expression level in tumor tissues was significantly higher than those in the corresponding normal tissues (*P* < 0.001). Next, we explored the correlation between AFAP1-AS1 expression and the clinic-pathological factors of patients with NSCLC. In general, AFAP1-AS1 level was associated with TNM stage and tumor size. Patients with advanced TNM stage (III/IV) or tumor size larger than 30 mm were associated with higher AFAP1-AS1 expression, whereas patients with local TNM stage (I/II) or tumor size smaller than 30 mm were associated with lower AFAP1-AS1, respectively (Fig. 1b, P =0.046) (Fig. 1c, P =0.039). Furthermore, we divided the samples into relative high (above the mean, *n* = 46) and relative low (below the mean, n = 46) AFAP1-AS1 expression groups according to the median value of AFAP1-AS1 levels. Chi-square test was then performed to evaluate clinic-pathological factors between the two groups. As shown in Table 1, relative AFAP1-AS1 level was also correlated to tumor size (*P* < 0.001). No relationship between AFAP1-AS1 expression and other clinical factors, e. g. sex (male, female), patients’ age (≤60, > 60), histological grade (middle or low, high), histological classification, lymph node metastasis (negative, positive) or history of smoking (ever, never) was found in our study.

### Over-expression of AFAP1-AS1 level reveals a poor overall survival time (OS) of patients with NSCLC and could be regarded AS an independent predictor for OS

To determine the relationship between AFAP1-AS1 expression and NSCLC patients’ prognosis, we attempted to evaluate the correlation between AFAP1-AS1 expression and clinical outcomes. Kaplan–Meier analysis and log-rank test were used to evaluate the effects of AFAP1-AS1 expression and the clinicopathological characteristics on overall survival (OS). The median survival time for low AFAP1-AS1 expression groups was 43.989 ± 2.238 months, while that for high AFAP1-AS1 expression groups was only 30.505 ± 2.288 months. As shown in Fig. 1d, over-expression of AFAP1-AS1 predicted a poor prognosis in patients with NSCLC (*P* < 0.001). To further validate this result, we analyzed RNA-Seq data (from TCGA: The Cancer Genome Atlas) of lncRNAs of NSCLC were from TANRIC [23] (http://ibl.mdanderson.org/tanric/_design/basic/index.html). As shown in Fig. 1e, AFAP1-AS1 was significantly up-regulated in NSCLC tissues from TCGA data, both in lung adenocarcinoma (LUAD) and Lung squamous cell carcinoma (LUSC).

To further confirm the prognostic role of AFAP1-AS1 in NSCLC patients, the univariate and multivariate survival analysis (Cox proportional hazards regression model) were performed. Univariate analysis identified three prognostic factors: histological grade (middle or low, high), TNM stage (I/II, III/IV) and AFAP1-AS1 expression. Multivariate analysis further revealed that AFAP1-AS1 expression could be regarded as an independent predictor for overall survival in patients with NSCLC (*P* < 0.001), as well as TNM stage (*P* = 0.001) and histological grade (*P* = 0.048) (Table 2).

**Table 2:** Univariate and multivariate analysis of clinic pathologic factors for overall survival in 92 patients with NSCLC

Risk factors	Univariate analysis			multivariate analysis		
	HR^a^	p value	95% CI	HR	*p* value	95% CI
AFAP1-AS1 expression	1.057	< 0.001**	1.039~ 1.075	1.046	< 0.001**	1.027~ 1.064
TNM stage (I/II, III/IV)	2.826	< 0.001**	1.688~ 4.730	2.618	0.001**	1.494~ 4.588
Histological grade (middle or low, high)	1.812	0.020*	1.100~ 2.986	1.697	0.048*	1.004~ 2.871
Tumor size (≤3 cm, > 3 cm)	1.561	0.086	0.939~ 2.595			
Histological classification (SCC, AD or another)	1.187	0.487	0.731~ 1.928			
Age (≤60, > 60)	0.941	0.806	0.580~ 1.527			
N (negative, positive)	1.395	0.185	0.853~ 2.281			
History of smoking (ever, never)	1.17	0.525	0.721~ 1.900			
Sex (male, female)	1.236	0.39	0.762~ 2.006			

^a^HR hazard ratio

*p < 0.05

**p < 0.01

### Knockdown of AFAP1-AS1 impaired NSCLC cells proliferation and induced cell cycle arrest in vitro

**Fig. 2:**
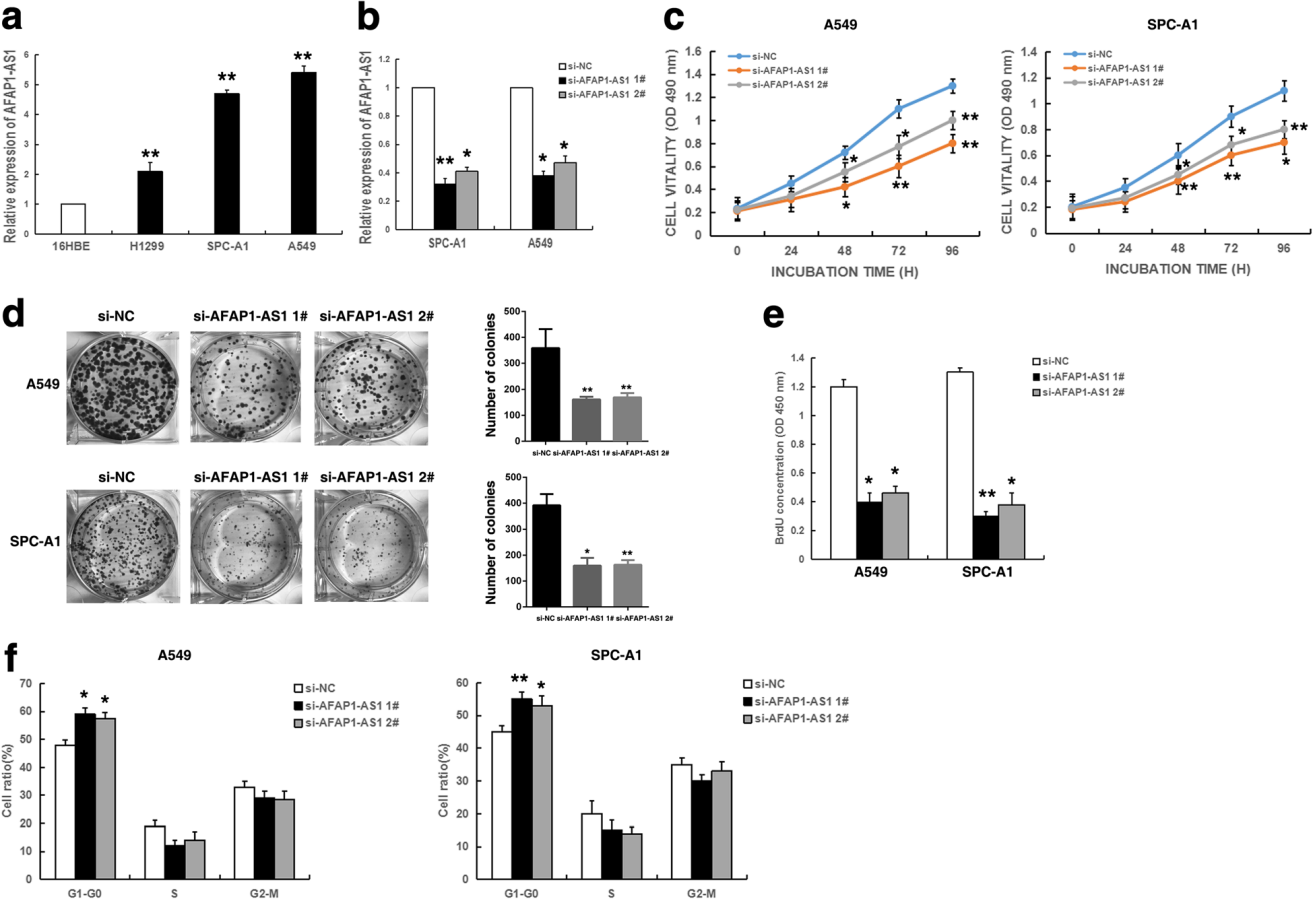
AFAP1-AS1 regulates NSCLC cell proliferation in vitro. **a** Analysis of AFAP1-AS1 expression levels in NSCLC cell lines compared with a normal bronchial epithelial cell line (16HBE) using qRT-PCR. **b** The relative expression levels of AFAP1-AS1 in A549 and SPCA1 cells transfected with si-NC or si-AFAP1-AS1 (si-AFAP1-AS1 1# and si-AFAP1-AS1 2#) were measured using qPCR. **c** MTT assays were performed after transfection to determine the cell viability of the transfected A549 and SPCA1 cells. **d** Colony-forming assays were conducted after transfection to determine the proliferation of the transfected A549 and SPCA1 cells. The colonies were counted and captured. **e** BrdU assays were used to detect the cell proliferation after transfection, respectively. **f** After transfection, the cell cycle stages of A549 and SPCA1 cells were analyzed by flow cytometry. The bar chart represents the percentages of cells in the G1/G0, S, or G2/M phases, as indicated.*, *P* < 0.05, and **, *P* < 0.01

To gain insight into the functional role of AFAP1-AS1 in NSCLC cells, we first performed qRT-PCR analysis to detect the AFAP1-AS1 expression in diverse human NSCLC cell lines and a normal human bronchial epithelial cell line (16HBE). As shown in Fig. 2a, A549 and SPCA1 cells expressed higher AFAP1-AS1 levels compared with 16HBE. To investigate the potential role of AFAP1-AS1 in NSCLC cells, we uesd two siRNAs to transfect these two cell lines and silence AFAP1-AS1 expression. At 48 h post-transfection, qRT-PCR analysis revealed that AFAP1-AS1 expression was knocked down by both in A549 and SPCA1 cells (Fig. 2b).

Then, MTT assays showed that knockdown of AFAP1-AS1 expression significantly inhibited cell viability and cell proliferation both in A549 and SPCA1 cell lines compared to control cells (Fig. 2c). Similarly, results of colony-formation assays revealed that a decrease in AFAP1-AS1 expression also greatly attenuated the colony-forming ability of A549 and SPCA1 (Fig. 2d). Then BrdU assays demonstrated that AFAP1-AS1 knockdown had a significant repression on NSCLC cell proliferation (Fig. 2e). Next, flow cytometric analysis was performed to further examine the effect of AFAP1-AS1 on the proliferation of NSCLC cells by altering cell cycle progression. The results revealed that the cell cycle progression of si-AFAP1-AS1 cells was significantly stalled at the G1–G0 phase compared with cells transfected with si-NC, both in A549 and SPCA1 cells (Fig. 2f).

### Knockdown of AFAP1-AS1 inhibits NSCLC cell tumorigenesis in vivo

**Fig. 3:**
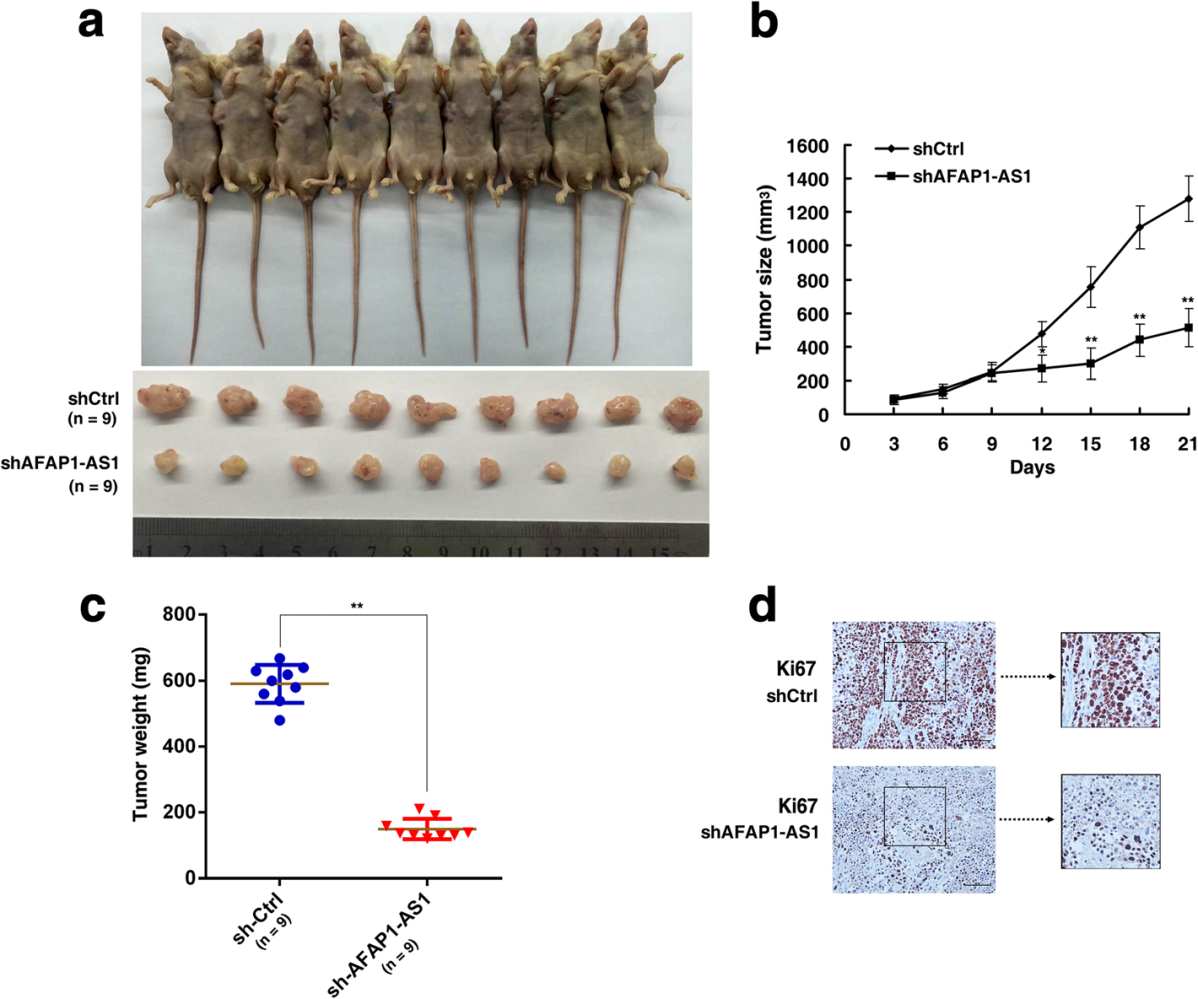
AFAP1-AS1 regulates cell growth in vivo. **a** Empty vector or sh-AFAP1-AS1 was transfected into A549 cells, which were then injected individual into nude mice (n = 9) (control on left, shAFAP-AS on right). **b** The tumor volumes were calculated every three days after injection. **c** The tumor weights are represented as the mean tumor weights ± SD. **d** The tumor sections were examined using IHC staining with antibodies against Ki-67. *, *P* < 0. 05, and **, *P* < 0.01

To further prove the role of AFAP1-AS1 in in vivo, we used a xenograft mouse model. A549 cells stably transfected with sh-AFAP1-AS1 or an empty vector were subcutaneously injected into nude mice (control on left, shAFAP-AS on right). The results showed that tumors grown from AFAP1-AS1 stable knockdown cells were smaller than tumors grown from control cells (Fig. 3a and b). Up to twenty-one days after injection, the average tumor weight in the shAFAP1-AS1 group was markedly lower than that in the control group (Fig. 3c). Moreover, IHC staining showed that tumor tissues collected from the AFAP1-AS1 knockdown group had fewer Ki67- positive cells (Fig. 3d).

### AFAP1-AS1 epigenetically silences p21 transcription by binding to EZH2

**Fig. 4:**
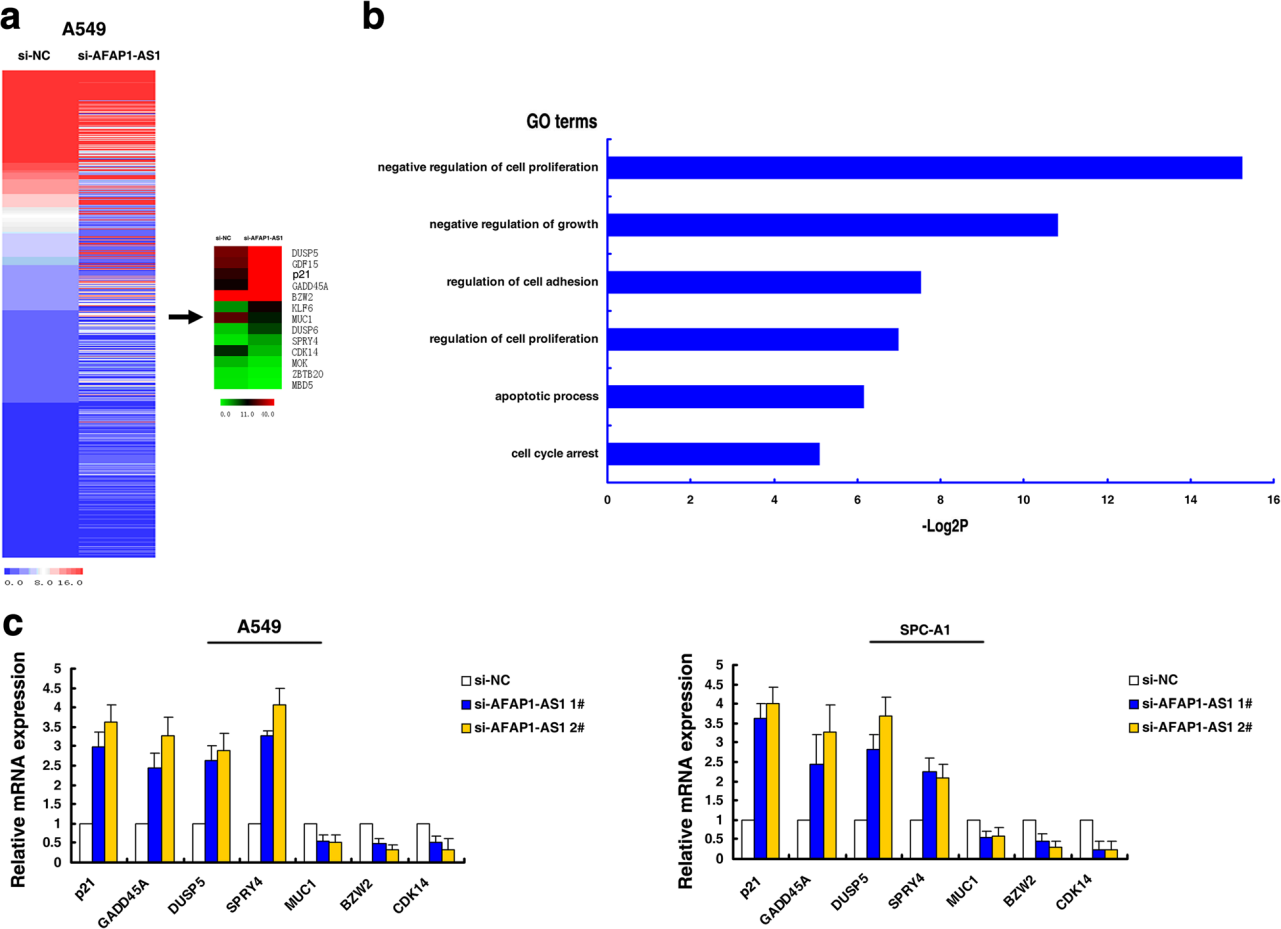
RNA-seq assay after AFAP1-AS1 knockdown in A549 cells. **a** Mean-centered, hierarchical clustering of 591 transcripts altered (≥2-fold change) in si-NC-treated cells and siRNA-AFAP1-AS1 treated A549 cells. **b** Gene Ontology analysis for all genes with altered expressions. **c** The altered mRNA levels of genes were selectively confirmed by qRT-PCR in knockdown AFAP1-AS1

Although AFAP1-AS1 has exhibited oncogenic property in various type of cancer, however, the exact molecular mechanism and global genes which were mediated by AFAP1-AS1 remains unclear. To probe the AFAP1-AS1-associated pathway on an unbiased basis in NSCLC, we assessed the gene expression profiles of A549 cells that were knockdown of AFAP1-AS1. We applied RNA transcriptome sequencing from control or siRNAs against AFAP1-AS1. A common set of 347 mRNAs showed ≥2-fold increased expression in AFAP1-AS1-depleted cells and silencing AFAP1-AS1 also reduced the abundance (≤ 2-fold) of 244 genes (Fig. 4a, Additional file 2: Table S2). Gene ontology (GO) analysis showed that the most significantly overrepresented biological processes included pathways involved in cell proliferation and cell cycle et al. (Fig. 4b). Several genes that contribute to tumorigenesis were selected and confirmed by qPCR assays (Fig. 4c).

**Fig. 5:**
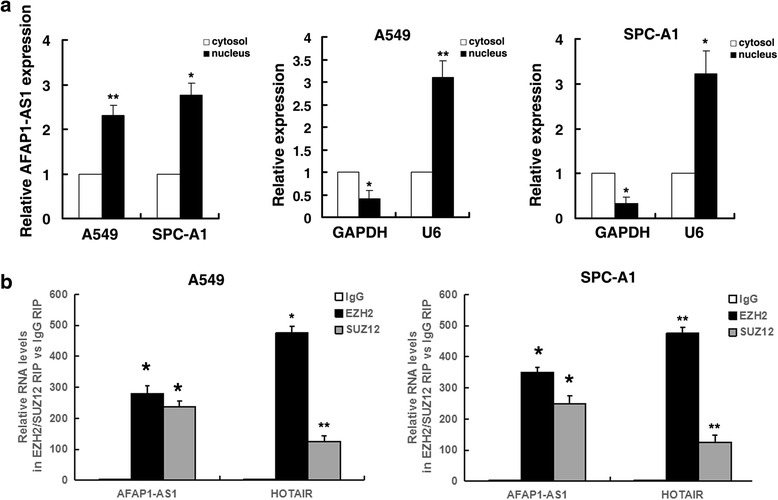
AFAP1-AS1 could directly bind to EZH2. **a** After nuclear and cytosolic separation, RNA expression levels were measured by qRT-PCR. GAPDH was used as a cytosol marker and U6 was used as a nucleus marker. **b** RIPs experiments for EZH2, SUZ12 were performed and the coprecipitated RNA was subjected to qRT-PCR for AFAP1-AS1

Recent studies have reported that a significant number of lncRNAs have been shown to function in cooperation with chromatin modifying enzymes to promote epigenetic activation or silencing of gene expression [24]. Previous studies have concluded that approximately 20% of all human lncRNAs can physically associate with Polycomb repressive complexe 2 (PRC2), suggesting that lncRNAs may have a general role in recruiting polycomb-group proteins to their target genes [25]. Enhancer of zeste homolog 2 (EZH2), the catalytic subunit of the PRC2, could epigenetically repress transcription of specific genes [26]. In addition, aberrations in EZH2 are closely related to carcinogenesis [27]. Thus, we hypothesized that AFAP1-AS1 might regulate gene expression in such a manner. To test this, first, we found a considerable increase in AFAP1-AS1 expression in the nucleus versus the cytosol (Fig. 5a), GAPDH was used as a cytosol marker and U6 was used as a nucleus marker, thus suggesting that AFAP1-AS1 may play a major regulatory function at the transcriptional level. Then to validate the possibility interaction between AFAP1-AS1 and EZH2, RNA immunoprecipitation (RIP) assays showed that the endogenous AFAP1-AS1 was enriched in the anti-EZH2 RIP fraction relative to the input compared to the IgG fraction. Moreover, using an antibody specific to SUZ12, another member of the PRC2 complex, we also observed that AFAP1-AS1 was enriched in the anti-SUZ12 RNA-IP fraction, both in A549 and SPC-A1 cells (Fig. 5b). The endogenous lncRNA HOTAIR, which binds PRC2, was used as positive control [28].

**Fig. 6:**
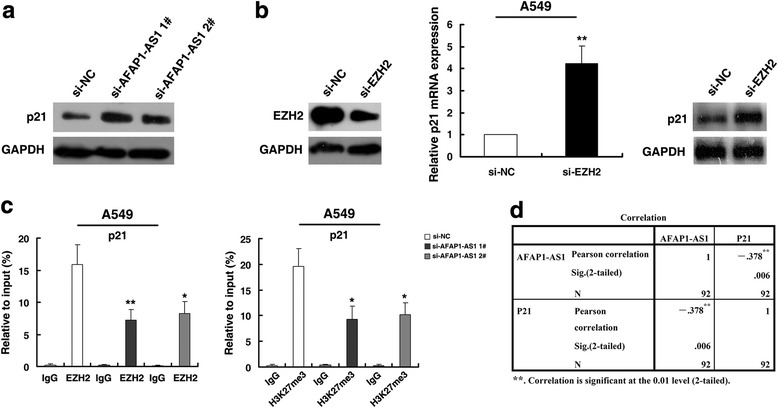
AFAP1-AS1 epigenetically silences p21 transcription by binding to EZH2. **a** Western blot analysis of p21 protein level in A549 after AFAP1-AS1 knockdown. **b** QPCR and Western blot analysis of p21 mRNA and protein level in A549 after EZH2 knockdown. **c** ChIP-qPCR of EZH2/H3K27me3 of the promoter region of p21 genes locus after siRNA treatment targeting AFAP1-AS1 in A549 cells. Antibody enrichment was quantified relative to the amount of input DNA. Antibody directed against IgG was used as a negative control. **d** AFAP1-AS1 were negatively correlated with p21 expression in tissues. *, *P* < 0.05, and **, *P* < 0.01

RNA-Seq found that knockdown of AFAP1-AS1 could significantly decrease a series of genes which promoting proliferation. Among these genes, p21 attracted our attention because of its established tumor suppressor’s role in tumorigenesis and involved in cancer cell cycle. Importantly, aberrant methylation in promoter regions of p21 has been linked to downregulation of p21 in cancer cells [29]. In addition, PRC2-mediated histone methylation contributes to the repression of p21 [30]. Hence, we chosed p21 for further investigation. As shown in Fig. 6a, western blot analysis showed that the protein expression levels of p21 after knockdown AFAP1-AS1. Importantly, qRT-PCR and western blot analyses show that EZH2 knockdown could induce expression levels of p21 mRNA and protein (Fig. 6b).

We next investigated whether EZH2 could bind to the promoter regions of p21 which mediated by AFAP1-AS1, we performed chromatin immunoprecipitation (ChIP) assays. The results showed that EZH2 could bind to the p21 promoter regions, and knockdown of AFAP1-AS1 reduced EZH2-mediated H3K27me3 trimethylation (Fig. 6c). Moreover, qRT-PCR analysis found that p21 was significantly downregulated in 92 pairs of NSCLC tissues. Further analysis demonstrated that AFAP1-AS1 were negatively correlated with p21 expression (Fig. 6d). These results suggest that AFAP1-AS1 could promote NSCLC cell growth partly through epigenetically silencing p21 transcription by binding to EZH2.

## Discussion

Recently, it is becoming clear that mammalian genomes encode thousands of lncRNAs [13, 31]. To date, increasing evidence links dysregulation of lncRNAs to diverse human diseases especially in tumorigenesis [14, 32]. Our previous studies also revealed that lncRNA CCAT1 could regulate cell proliferation and migration in esophageal squamous cell carcinoma [33]. And TUG1 play an important role in NSCLC [34]. In this study, we discovered an lncRNA AFAP1-AS1 whose elevated expression in NSCLC tissues was highly associated with the tumor suppressor p21. Moreover, we showed that increased AFAP1-AS1 expression was associated with a poor prognosis and shorter survival time in NSCLC patients. Our gene expression and functional data strongly support the potential utility of AFAP1-AS1 as a biomarker and as a therapeutic target for NSCLC.

AFAP1-AS1 is the first identified lncRNA that is overexpressed in primary BE and EAC tissues as well as in EAC cell lines [15]. Besides, knockdown of LncRNA AFAP1-AS1 in GBC cells inhibited EMT by upregulating the E-cadherin and downregulating the transcription factor Twist1 and Vimentin [20]. Furthermore, AFAP1-AS1 can promote HCC development through upregulation of RhoA/Rac2 signaling and provide a potential therapeutic target for HCC [18]. Recently, Deng J et al. found that AFAP1-AS1 may be an independent prognostic marker for NSCLC [35]. Collectively, these studies have revealed that AFAP1-AS1 has an important role in tumor carcinogenesis and acts as an oncogenic lncRNA. But, the biological functions of AFAP1-AS1 in the control of NSCLC tumorigenesis have not been well characterized. Especially, the molecular mechanism and global genes that were mediated by AFAP1-AS1 remain unknown. Our study suggested that AFAP1-AS may have an oncogenic function in NSCLC progression.

Generally, lncRNAs exert their function through interacting with various RNA binding proteins (RBPs) and leading to inactivation or activation of gene expression via chromosome reprogramming, DNA methylation, RNA decay and histone protein modification [36, 37]. HOTAIR is one of the most studied lncRNAs involved in chromatin modification, which can recruit PRC2 genome-wide to alter H3K27 methylation and gene expression patterns [28, 38]. It is evident that more than 20% of lncRNAs are bound by PRC2 in various cells, and silence downstream target genes [25]. It is well documented that PRC2 is a critical regulator of histone modification, which catalyzes the trimethylation of H3K27 to mediate gene silencing. Recent findings implicate that PRC2 is an important driver of tumor development and progression by suppressing various key genes. EZH2 is a core subunit of PRC2 complex that could catalyze the trimethylation of lysine residue 27 of histone 3 (H3K27me3), which is over-expressed in multiple cancers [39]. In this study, RIP assays confirmed that AFAP1-AS1 could bind to EZH2 in NSCLC cells.

RNA-Seq data showed that p21 might be downstream mediators of AFAP1-AS1. P21, is one of the most crucial member of the cyclin-dependent kinase (CDK) inhibitor family for G1/S transition [40–42]. Additionally, substantial evidence from biochemical and genetic studies indicates that p21 acts as a master molecule of multiple tumor suppressor pathways for promoting anti-proliferative activities in cancer cells [43, 44]. Notably, Our RNA-seq data found that p21 was significantly upregulated after AFAP1-AS1 knockdown. In addition, ChIP assays validated that knockdown of AFAP1-AS1 resulted in the loss of H3K27 trimethylation and EZH2 binding to the genomic loci of p21, confirming that p21 was a bona target of AFAP1-AS1/EZH2-regulated genes. Our findings demonstrated that AFAP1-AS1 promotes lung cancer cell proliferation by epigenetically repressing p21 expression.

## Conclusions

In summary, we had evidenced that expression of the lncRNA AFAP1-AS1 is upregulated in the NSCLC tissues and cells, and increased AFAP1-AS1 is associated with poor prognosis of NSCLC patients. The loss of AFAP1-AS1 inhibited NSCLC cell proliferation in vitro and suppressed tumor growth in vivo. Furthermore, AFAP1-AS1-mediated oncogenic effects occurred partially through epigenetic suppression of p21 expression by binding to EZH2. Our findings increase our understanding of the pathogenesis and progression of NSCLC and may facilitate the development of lncRNA-directed diagnostics and therapeutics in NSCLC.

## Additional files

Additional file 1: **TableS1.** The list of primers and the sequence of siRNAs. (XLS 8 kb)

Additional file 2: **TableS2.** mRNAs increased abundance (≥2-fold) in AFAP1-AS1 knockdown A549 cells. (XLS 125 kb)

## Abbreviations

ChIP: Chromatin immunoprecipitation
H3K27me3: histone 3 lysine 27 trimethylation
IHC: Immunohistochemical
lncRNAs: Long noncoding RNAs
NSCLC: non–small cell lung cancer
qRT-PCR: Quantitative real time polymerase chain reaction
RIP: RNA immunoprecipitation
TCGA: The Cancer Genome Atlas
